# Research on Satellite Network Traffic Prediction Based on Improved GRU Neural Network

**DOI:** 10.3390/s22228678

**Published:** 2022-11-10

**Authors:** Zhiguo Liu, Weijie Li, Jianxin Feng, Jiaojiao Zhang

**Affiliations:** Communication and Network Laboratory, Dalian University, Dalian 116622, China

**Keywords:** satellite network, flow forecast, attention mechanism, neural network

## Abstract

The current satellite network traffic forecasting methods cannot fully exploit the long correlation between satellite traffic sequences, which leads to large network traffic forecasting errors and low forecasting accuracy. To solve these problems, we propose a satellite network traffic forecasting method with an improved gate recurrent unit (GRU). This method combines the attention mechanism with GRU neural network, fully mines the characteristics of self-similarity and long correlation among traffic data sequences, pays attention to the importance of traffic data and hidden state, learns the time-dependent characteristics of input sequences, and mines the interdependent characteristics of data sequences to improve the prediction accuracy. Particle Swarm Optimization (PSO) algorithm is used to obtain the best network model Hyperparameter and improve the prediction efficiency. Simulation results show that the proposed method has the best fitting effect with real traffic data, and the errors are reduced by 26.9%, 37.2%, and 57.8% compared with the GRU, Support Vector Machine (SVM), and Fractional Autoregressive Integration Moving Average (FARIMA) models, respectively.

## 1. Introduction

With the continuous development of Internet information technology, the current means of communication are gradually becoming dominated by satellites. Satellite networks have wide coverage, high communication quality, and support for various services, and they are playing an increasingly important role in the fields of global communication, navigation and positioning, environmental and disaster detection, and military applications [1]. For these, traffic information is an important feedback index of network load state. Network traffic management is needed to prevent network congestion and improve the routing effect. At the same time, in practical application, due to the limited bandwidth resources of satellite networks, the traffic planning problem has become a key component of a satellite communication network. With the increasing number of users of satellite communication networks, the network traffic presents great uncertainty, which causes the traditional traffic planning methods to have serious lag and discomfort. As a result, it directly leads to an imbalance in network resource allocation and aggravates the congestion on the communication link, which leads to the failure rate, delay, and QoS of the network being difficult to guarantee. Therefore, in recent years, research on flow planning has gradually changed into a predictive dynamic planning scheme. Fast and accurate traffic forecasts for satellite networks is of great significance to the development of satellite communication networks.

Compared with terrestrial network communication, the communication transmission delay of satellite communication is large [2]. Because of the large transmission delay, the traffic allocation scheme needs to be based on the traffic prediction value of the next time in the satellite network. At the same time, in the space–world integrated network, part of the traffic on the satellite is uploaded from the ground. Because this part of the traffic is not stable in the time domain, it may make the routing result invalid, and the network will be congested and unable to respond to the service in real time. Through the forecast of satellite network traffic, we can grasp the changing characteristics and trends of network traffic in advance. At the same time, according to the traffic load of each satellite, the path selection can be planned to avoid local congestion in the network and improve the routing strategy efficiency of the network.

Aiming at the problems of low prediction accuracy and low efficiency of current satellite network traffic prediction methods, this paper fully considers the characteristics of self-similarity and long correlation of satellite network traffic data and proposes an improved GRU neural network satellite network traffic prediction model. Based on the traditional GRU neural network, this paper integrates the attention mechanism to form a new neural network model, which pays attention to the importance of traffic data and hidden state. It not only learns the time-dependent characteristics of input sequences but also mines the interdependent characteristics of data sequences, thus improving the accuracy of traffic prediction. At the same time, the particle swarm optimization (PSO) algorithm is used to determine the optimal super-parameter combination of the model, which makes the model have higher prediction efficiency in traffic forecasting.

The contributions of this paper are as follows:

(1) In this paper, the correlation characteristics of satellite network traffic are fully considered, and the nonlinear time dynamic correlation is obtained by using a gating unit to avoid gradient disappearance or gradient explosion during training.
(2) In the coding and decoding stages of the GRU network, an attention mechanism is introduced, and multiple intermediate vectors are added to uniformly process the time series and input information of the intermediate vectors at the current movement.
(3) Particle swarm optimization algorithm is used to adjust the hyperparameters of the neural network.

The organizational structure of this paper is as follows: The first part introduces the current research status of traffic forecasting at home and abroad. The second part introduces the definition of the satellite network traffic prediction problem and the overall framework of the Attention-GRU (AT-GRU) satellite network traffic prediction model proposed in this paper. The third part introduces the realization principle of each part of the model, explains the coding and decoding unit design module of the new prediction model in detail, and introduces the determination method of the new model parameters and the loss function. In the fourth part, the simulation comparison is carried out. By comparing it with several commonly used prediction models, it is shown that the new model has good prediction performance and high prediction accuracy. The fifth part is the summary and prospect, which summarizes the contents of this paper and points out the areas that can be further improved and optimized in the current research results.

## 2. Literature Review

In recent years, satellite network traffic forecasting methods have emerged endlessly, and the forecasting accuracy and efficiency of the different forecasting methods are different. The prediction models can be roughly divided into traditional mathematical–statistical fitting models and popular prediction models based on machine learning neural networks.

Markov model and time series model are commonly used in mathematical fitting models. Yan Z et al. analyzed the disadvantages of the usual Poisson traffic model and proposed a deterministic Markov modulation process model [3] to simulate satellite network traffic. The process of traffic acquisition, storage, and transmission was transformed into a queuing model, some closed expressions of service quality indicators were derived, and the validity of the theory was verified. Dong Y et al. put forward the Autoregressive Moving Average Model (ARMA) to forecast satellite network traffic [4], and the current traffic sampling value is represented by the weighted sum of several historical traffic sampling values. Chen et al. introduced the geographic longitude of the satellite and the transit time of traffic to establish a mathematical model and proposed a forecasting algorithm based on the proxy model to forecast the traffic volume in the satellite coverage area [5]. Although FARIMA can obtain the long-term and short-term correlation characteristics of network traffic data itself, the model is highly complex and its prediction accuracy is not high [6]. Traditional mathematical–statistical fitting models have poor prediction accuracy for current network traffic and lack good description ability for satellite long-term correlation characteristics, so they cannot be well used in satellite network traffic prediction.

In recent years, with the rapid development of artificial intelligence, neural networks are widely being used in satellite network traffic forecasting. In recent years, many experts and scholars have been using neural networks and deep learning to predict satellite network traffic. Compared with the traditional mathematical–statistical fitting model, the neural network model has a strong nonlinear mapping ability, generalization ability for complex network systems, and strong self-learning and self-organizing ability [7]. Zhou W et al. proposed a new model (SSA-AWELM) [8] which combines singular spectrum analysis (SSA) with AdaBoost weighted extreme learning machine (AWELM). SSA was developed to decompose the original data into three components: trend, periodicity, and residue. AWELM was developed to seperately predict every component. The three prediction results are added up to the final result. Fan et al. proposed combining Recurrent Neural Network (RNN) and GRU into a new network model [9] and applying it to network traffic prediction. A large number of experimental results show that the prediction result of this model is close to the real value. Vinchoff C et al. proposed a nonlinear GCN-GAN network model [10]. This model combines GCN and GAN, extracts the complex characteristics of network traffic through Graph Convolutional Neural Network (GCN), and uses Generative Adversarial Networks (GAN) to model the data structure. The proposed model realizes the prediction of burst traffic. Ming et al. put forward a new prediction model, LA-ResNet [11]. The innovation of this model is that an attention mechanism is added, but the disadvantage of this model is its complicated structure. Network traffic data is a kind of complex data with multiple characteristics, and the prediction accuracy of common models is not high. For neural networks, the more super-parameters there are, the larger the network will be and the more complex the model. To improve the prediction accuracy of the model and reduce the complexity of the model, Wang S et al. proposed a traffic prediction strategy to improve the LSTM network with a genetic algorithm [12]. In this scheme, LSTM is used to extract the time characteristics of network traffic, a genetic algorithm is used to get the hyperparameters of the proposed network, and finally, the proposed new network traffic prediction model is used to predict the network traffic. Nikesh AY et al. compared SVM, MPL, and MLPWD in the same network environment [13]. The simulation results show that the prediction effect of SVM is better than MPL and MLPWD. Liu D et al. proposed a new graph convolution network model [14], which considered the static spatial dependence of traffic and the influence of dynamic traffic on spatial relationships. Simulation results show that the prediction error of the proposed scheme is small. Sudhakaran S et al. proposed a traffic forecasting method using a deep neural network to model traffic [15]. It is realized by treating the data flow as a tensor and then transmitting it to the convolutional neural network. Finally, the results of the proposed network were verified using the Telecom Italia data set. Li N et al. proposed a neural network traffic prediction algorithm based on transfer learning [16]. Combined with transfer learning, the problem of insufficient online traffic data is solved. Experiments show that the proposed scheme can reduce the error of traffic prediction.

## 3. Definition and Model of the Satellite Traffic Forecast Problem

**Figure 1:**
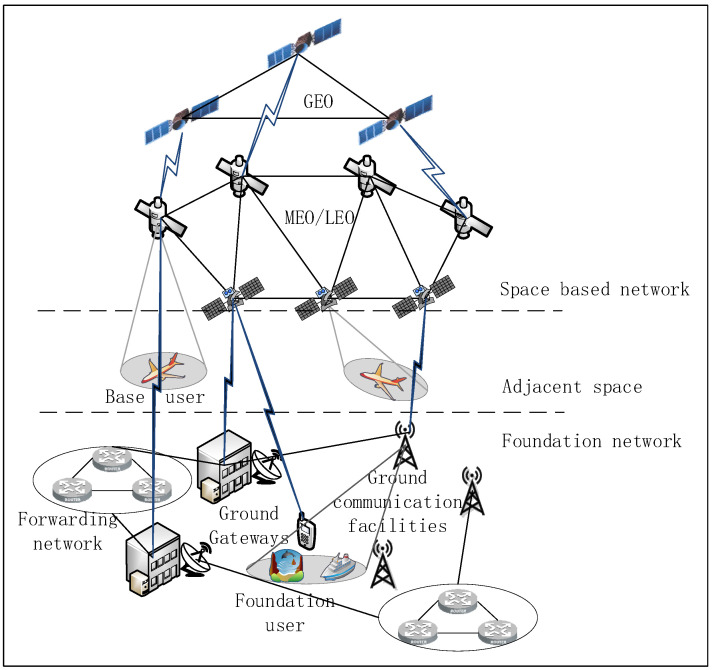
Satellite communication model diagram.

Figure 1 shows a system structure diagram of satellite communication. A satellite communication system consists of the space-based network, adjacent space and the ground-based network. The space-based network consists of Geosynchronous Earth Orbit (GEO), Middle Earth Orbit (MEO), and Low Earth Orbit (LEO), which mainly realize global coverage and broadband access. Adjacent space is composed of some aircraft, which mainly realize the function of edge service. The ground-based network is composed of ground gateways, network control centers, and satellite control centers, and is mainly responsible for network services in business-intensive areas. The terminal is a device that inputs programs and data to the computer or receives the processing results output from the computer via communication facilities. Satellite terminals can be divided into handheld terminals, portable terminals, vehicle-mounted terminals, etc., according to terminal types. Each satellite terminal is responsible for managing terminal connections in multiple systems. The terminal will be connected to the default gateway station of its beam when accessing the satellite network for communication. In the satellite uplink, the traffic generated on the ground is uploaded to the satellite, which then forwards it to the ground gateway station. In the satellite downlink, ground gateway stations and data centers complete traffic monitoring [17]. The traffic of all satellites comes partly from other satellites and partly from the ground network.

**Figure 2:**
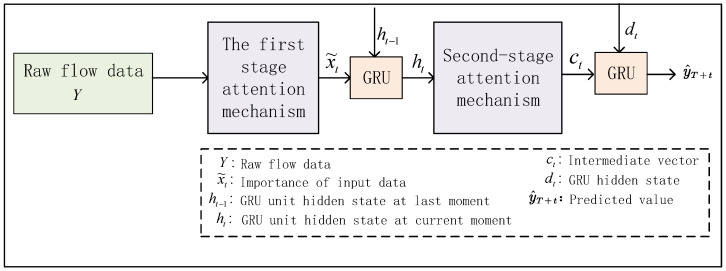
Traffic prediction model of AT-GRU satellite network.

Network traffic prediction can guarantee high-quality communication, so it is widely used in many satellite applications. Satellite traffic has complex characteristics such as self-similarity and long correlation. Different from the terrestrial network, the available resources of the satellite network are more limited, and the topological structure of the satellite network changes from time to time. The satellite traffic prediction algorithm must take into account both accuracy and efficiency. Most ground network prediction models have high computational complexity. If the ground network traffic prediction model is directly applied to the satellite, it will increase the burden on the satellite. In this paper, a new neural network satellite network traffic prediction model with an attention mechanism is proposed, which can be applied to satellite network traffic prediction in terms of prediction accuracy and operation efficiency. The flow forecast model diagram is shown in Figure 2. The flow forecast mathematics is set as follows:

Given satellite network traffic data *Y*, where $Y=\left(y_{1},y_{2},\ldots y_{T}\right)$ represents the target sequence within the length of the window *T*. Based on the given time series information, the goal of satellite network traffic prediction is to predict the value of the time series *Y* before the *t* timestep, which can be expressed as follows

(1) $${\hat{y}}_{T+t}=F\left(y_{1},y_{2},\ldots ,y_{T}\right),$$

where ${\hat{y}}_{T+t}$ represents the predicted value and F(⋅) represents the nonlinear mapping function.

The overall structure model of this paper is shown in Figure 2. Based on the traditional GRU coding and decoding structure, this paper integrates the attention mechanism. The traditional coding–decoding structure must compress all the input information into fixed-length vectors. Using this simple fixed-length coding to represent longer and more complex inputs often leads to the loss of input information. The attention model allows the decoder to access the output generated by all encoders to overcome the above shortcomings. Its core idea is that all the outputs of the encoder are weighted and combined, and then input into the decoder at the current position to influence the output of the decoder. By weighing the output of the encoder, the alignment between the input and the output can be realized, and at the same time, more information about the original data can be used. The attention module can automatically learn the weight to capture the correlation between the hidden state of the encoder and the hidden state of the decoder.

Attention mechanism, as a milestone in deep learning research, can adjust the original input data according to different needs, and get new input data more in line with the current model. The purpose of this paper is to improve the GRU unit by introducing an attention mechanism, which is to improve the model’s acquisition of historical time series information, and integrate the historical time series information into memory cells, so that the whole model can obtain more historical flow information, and extract more helpful information for future prediction for prediction and analysis, thus improving the overall prediction accuracy. The combination of the attention mechanism and GRU can learn the spatial relationship between input variables in the coding stage, and then pay attention to the importance of different input sequences. In the decoding stage, the attention mechanism is introduced to select the hidden state of the encoder, and then the attention weight of the hidden state of the encoder is obtained, and finally, the network traffic prediction value is obtained.

## 4. Traffic Prediction Method of the AT-GRU Satellite Network

### 4.1. Design of Coding Unit Based on Attention Mechanism

**Figure 3:**
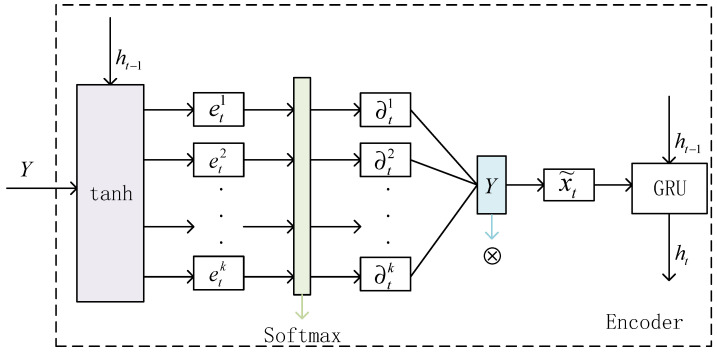
Coding stage.

To capture the long-term dependence on satellite traffic data, the encoder structure is based on the GRU unit [18,19]. GRU is a variant network form derived from the recurrent neural network RNN which incorporates a gating unit into the basic structure of RNN and controls the flow of information through the “gate” structure. It can encode the input sequence as a feature representation, and combined with the attention mechanism [20,21,22], the purpose of the encoding stage is to learn the spatial relationship between input variables. The encoding stage is shown in Figure 3.

Given the input sequence *Y*, GRU neural network is used to learn the nonlinear mapping function between the input sequence and the hidden state *h_t_* of the encoder at time *t*:

(2) $$h_{t}=f_{e}\left(h_{t-1},Y\right),\;$$

where $f_{e}\left(\cdot \right)$ stands for GRU unit. The GRU unit update process is summarized as follows:

(3) $$r_{t}=\sigma \left(W_{r}\left[h_{t-1},Y\right]+b_{r}\right),\;$$

(4) $$z_{t}=\sigma \left(W_{z}\left[h_{t-1},Y\right]+b_{z}\right),\;$$

(5) $${\tilde{h}}_{t}=\tanh\left(W_{\tilde{h}}\left[h_{t-1},Y\right]+b_{\tilde{h}}\right),$$

(6) $$h_{t}=(1-z_{t})\cdot h_{t-1}+z_{t}\cdot {\tilde{h}}_{t},$$

where $r_{t}$ is the reset gate, and how many previous states can be controlled by the reset gate that need to be memorized and stored; $z_{t}$ is the update gate, which is used to control how cells store information; $W_{r}$, $W_{z}$, $W_{\tilde{h}}$ represent the hidden layer weight; $b_{r}$, $b_{z}$, $b_{\tilde{h}}$ represent offset values, which are all parameters that need to be optimized for learning; · indicates point multiplication relation; $\left[h_{t-1},Y\right]$ indicates the concatenation of the currently input network traffic data and the previous hidden state; σ means activation function.

Combining the attention mechanism with GRU neural network, the spatial relationship between input variables can be adaptively learned. Given the Kth sequence $Y^{k}$ of input data, the attention mechanism in the first stage can be expressed as

(7) $$e_{t}^{k}=v_{e}^{T}\tanh\left(U_{e}\left[h_{t-1},Y^{k}\right]\right),$$

(8) $$\alpha_{t}^{k}=\frac{\exp\left(e_{t}^{k}\right)}{\sum_{i=1}^{n}\exp\left(e_{t}^{i}\right)},$$

where *h_t_*_−1_ represents the historical hidden state of the encoder, *v* and *U* are all parameters to be learned, and $e_{t}^{k}$ can be obtained by the tanh transformation. Attention weight (determined by the historical hidden state of the encoder and the current input value) is introduced to describe the importance of the kth input feature. Formula (8) ensures that all attention weights add up to 1.

At this point, Formula (2) can be updated to:


(9) $$h_{t}=f_{e}\left(h_{t-1},{\tilde{x}}_{t}\right).$$

By designing an encoder with an attention mechanism, we can pay attention to the importance of different input sequences, instead of treating all input sequences uniformly.

### 4.2. Design of Decoding Unit Based on Attention Mechanism

**Figure 4:**
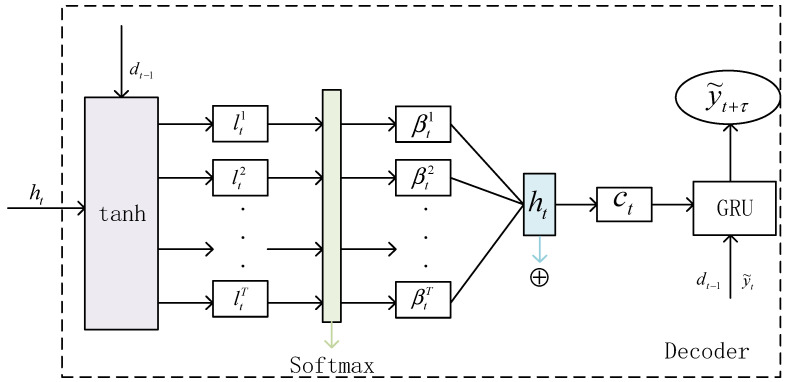
Decoding stage.

To simplify the model design, the decoding unit still adopts the GRU unit. In the decoding stage, the time attention mechanism and GRU neural network are combined to select and weight the hidden state *h_t_* of the encoder. In this way, the time relationship of the input sequence can be learned. The decoding stage is shown in Figure 4.

Assuming the hidden state *h_t_* of the encoder at the moment, the attention weight of the hidden state of the encoder can be expressed as

(10) $$l_{t}=v_{d}^{T}\tanh\left(W_{d}\left[d_{t-1},h_{t}\right]\right),$$

(11) $$\beta_{t}=\frac{\exp\left(l_{t}\right)}{\sum_{j=1}^{T}\exp\left(l_{t}^{j}\right)},$$

where *d_t_*_−1_ represents the historical hidden state of the previous decoder, v and W_t represent the parameters to be learned by the neural network, and *l_t_* can be obtained by the tanh transformation. β_t represents the importance of the hidden state of the *t*-th encoder. The intermediate quantity *c_t_* can be calculated as follows:

(12) $$c_{t}=\sum_{t=1}^{T}\beta_{t}h_{t}.$$

After obtaining the intermediate vector, combine it with the satellite network traffic data sequence $\left(y_{1},y_{2},\ldots ,y_{T}\right)$, as follows:

(13) $${\tilde{y}}_{t}=W\left[y_{T},c_{t}\right]+\tilde{b}.\;$$

The hidden state of the decoder at t time can be expressed as

(14) $$d_{t}=f_{d}\left(d_{t-1},{\tilde{y}}_{t}\right),\;$$

where $f_{d}\left(\cdot \right)$ stands for GRU unit, and $d_{t}$ can be updated to:

(15) $$r_{t}=\sigma \left(W_{r}\left[d_{t-1},{\tilde{y}}_{t-1}\right]+b_{r}\right),\;$$

(16) $$z_{t}=\sigma \left(W_{z}\left[d_{t-1},{\tilde{y}}_{t-1}\right]+b_{z}\right),\;$$

(17) $${\tilde{d}}_{t}=\tanh\left(W_{\tilde{d}}\left[d_{t-1}\cdot r_{t},{\tilde{y}}_{t-1}\right]+b_{\tilde{d}}\right),\;$$

(18) $$d_{t}=\left(1-z_{t}\right)\cdot d_{t-1}+z_{t}\cdot {\tilde{d}}_{t},\;$$

where *r_t_* is the reset gate, and how many previous states can be controlled by the reset gate that need to be memorized and stored; *z_t_* is the update gate, which is used to control how cells store information; *W_r_*, *W_z_*, $W_{\tilde{d}}$ represent the hidden layer weight; *b_r_*, *b_z_*, $b_{\tilde{d}}$ are offset values, which are all parameters that need to be optimized for learning; · indicates point multiplication relation; $\left[d_{t-1},{\tilde{y}}_{t-1}\right]$ represents the concatenation of the previous hidden state and the decoder input data; *σ* means activation function.

Get the network prediction value ${\hat{y}}_{T+t}$ at the *t* moment:

(19) $${\tilde{y}}_{T+t}=F_{ed}\left(y_{1},y_{2},\ldots ,y_{T}\right)=g\left(d_{t},c_{t}\right),\;$$

where g(⋅) is a linear change; the softmax function is selected in this paper.

### 4.3. PSO Algorithm for GRU Hyperparameter Selection Problem

There are many network hyperparameters in the construction of a neural network, such as Learning_rate, number of neurons, and epoch. These hyperparameters not only determine the fitting effect of the neural network but also affect the training effect of the model. To achieve the best effect on the network, a strategy should be adopted to determine the values of these hyperparameters. The PSO algorithm is an optimization method based on Swarm Intelligence. Its advantages lie in its simplicity, easy implementation, and profound intelligent background. It is suitable for both scientific research and engineering applications, and there are not many parameters to adjust. Combining the good global optimization ability of the PSO algorithm with a neural network can improve the generalization ability and learning performance of a neural network, thus improving the overall working efficiency of the neural network. In this paper, the values of hyperparameters are determined according to the results of the PSO algorithm, and the optimal structure of the network model is obtained. A particle of PSO represents a hyperparameter combination scheme. In this experiment, a particle is an array of [Number of neurons, Learning_rate, Epoch]. PSO is the process of calculating the fitness of each particle’s corresponding scheme and finding the most suitable scheme. The target function selected by PSO algorithm in this paper is the fitness function. In order to calculate the fitness value of each particle, fitness function takes the sum of errors between the predicted value and the real value; we set the best fitness function as:

(20) $$f=\frac{1}{N}\sum_{i=1}^{N}{\left(y_{i}-\tilde{y_{i}}\right)}^{2},\;$$

where *N* is the number of samples, $y_{i}$ is the actual value, and $\tilde{y_{i}}$ is the predicted value.

This paper applies the constraint condition of the algorithm, that is, the value range of the hyperparameters. Lecun Y et al.’ s research [23] provides the rules of hyperparameter configuration for neural networks. According to the above references, the initial population number is 20, the position range of each particle is [1, 150], [0.001, 0.15], [100, 700], that is, the number of neurons is set between [1, 150], the learning rate is set between [0.001,0.15], and the number of iterations is set at [100, 700]. The particle update formula is as follows:

(21) $$v_{i}=v_{i}+c1\cdot rand\cdot \left(pbest_{i}-a_{i}\right)+c2\cdot rand\cdot \left(gbest-a_{i}\right),$$

(22) $$a_{i}=a_{i}+v_{i}.$$

In which *v_i_* is the velocity of the particle, *a_i_* is the current position of the particle, i=1,2,…,N, *N* is the total number of particles in the particle swarm, *rand* is a random number, *pbest_i_* is the local optimal solution of the particle *i*, *gbest* is the global optimal solution of the particle swarm, that is, *gbest* is the best value among all *pbest_i_*, and c1 and c2 are learning factors. According to the research of Song Mengpei et al. [24], c1 and c2 in this paper are set c1=c2=2.

The Hyperparameter algorithm description of the PSO model is shown in Algorithms 1: Description of Hyperparameter algorithm of PSO optimization model.
**Algorithms 1:** Optimization algorithm of model hyperparameters based on PSO**Input:** Initialize parameters such as population size and iteration times; **Outputs:** Optimal hyperparameters of neural network; 1.     Procedure PSO; 2.     i=[Number of neurons,Learning_rate,Epoch] 3. **for** each particle *i*
**do** 4.        Initialize the velocity *v_i_* and position of particle *i* 5.        Evaluate particle *i* and set $pbest_{i}=a_{i}$ 6. **end for** 7. $gbest=\min\left\{pbest_{i}\right\}$; 8. **While** not stopping **do** 9.          **for** i=1 to N
**do** 10.                Update the velocity and position of the particle *i*; 11.              Evaluate particle i; 12.              **if** $fit\left(a_{i}\right)<fit\left(pbest_{i}\right)$ **then** 13.                   $pbest_{i}=a_{i}$ 14.              **end if** 15.              **if**
$fit\left(pbest_{i}\right)<fit\left(gbest\right)$ **then** 16.                   $gbest=pbest_{i}$ 17.              **end if** 18.       **end for** 19. **end while** 20. Print gbest 21. end procedure

### 4.4. Loss Function

The loss function can be used to show the difference between the predicted value and the real data. The loss function in this paper adopts the mean square error function:

(23) $$loss=\frac{1}{N}\sum_{i=1}^{N}{\left(y_{i}-{\tilde{y}}_{i}\right)}^{2},\;$$

where *N* represents the number of samples, *y_i_* represents the actual flow value, and ${\tilde{y}}_{i}$ represents the predicted flow value of the model proposed in this paper.

At the same time, Adam [25,26,27] optimizer is used to optimize the model parameters, and the loss function is reduced by updating the neuron weight matrix and offset value.

## 5. Results Simulation and Analysis

### 5.1. Description of the Data Set

**Figure 5:**
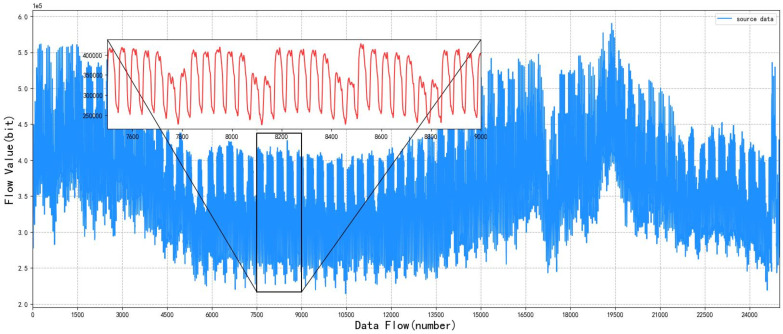
Original satellite network traffic data.

**Figure 6:**
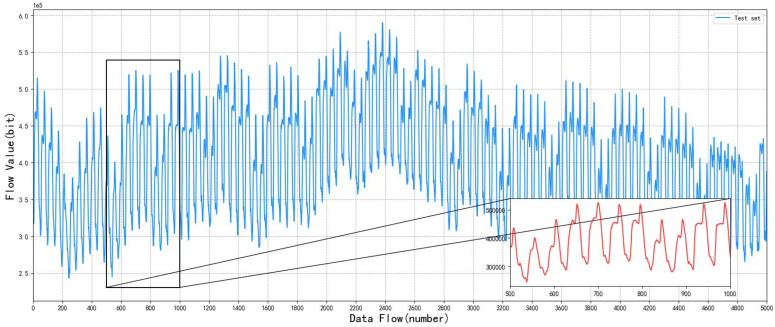
Test set of satellite network traffic data.

This simulation experiment uses OPNET and STK simulation software to simulate the information transmission network. From the Iridium constellation, we selected six satellites and two ground stations to simulate the integrated information network of heaven and earth. First, the data packets are routed through the satellite network to find the best path, then transmitted to the relay node, and finally arrive at the ground station. The *INT* node is the input end of the ground base station communication traffic in the analog network topology, and the node transmits the ground communication traffic into the satellite network. Four *rcv* nodes in the middle simulate the change of satellite topology with time. The *OUT* node is the output end of the communication traffic in the satellite network, which transmits the communication traffic back to the ground. The maximum number of hops from the output end to the input end is no more than three hops, which means that the link is disconnected. Each node will judge the connectivity of the current link according to its own position and the position of the surrounding nodes at the beginning of the simulation experiment, and save the connectable link as an optional link. Then, according to the optional links, the link with the minimum hop count is found to form the satellite network topology. The topology of the satellite network will change with time as the satellite nodes move in order to simulate the traffic characteristics of the integrated information network between heaven and earth, so this experiment intercepts some experimental data. In the experiment, 25,000 pieces of traffic data in the sampling period are taken as experimental data sets, and the data are divided into two parts: a training set and a testing set, with the training set accounting for 4/5 and the testing set accounting for 1/5. The original satellite network traffic data and test set obtained through simulation are shown in Figure 5 and Figure 6. The red lines in Figure 5 and Figure 6 are partial enlarged views of some sections.

### 5.2. Experimental Environment

The basic hardware environment based on the experiment is shown in Table 1. The experiment is based on the deep learning library of TensorFlow1.15. TensorFlow1.15 is an open source deep learning framework. TensorFlow can handle all kinds of neural networks conveniently. TensorFlow is used as the background program in the experiment, and matplotlib is used for visualization. We adopted TensorFlow1.15 version 1.15.

### 5.3. Evaluation Index and Parameter Setting of Simulation

#### 5.3.1. Evaluating Indicator

In this paper, the average absolute error MAE, root mean square error RMSE, and goodness of fit *R*^2^ are selected to evaluate the proposed prediction model:

(24) $$\mathrm{MAE}=\frac{1}{N}\sum_{i=1}^{N}\left|y_{i}-{\tilde{y}}_{i}\right|,\;$$

(25) $$\mathrm{RMSE}=\sqrt{\frac{1}{N}\sum_{i=1}^{N}{\left(y_{i}-{\tilde{y}}_{i}\right)}^{2}},\;$$

(26) $$R^{2}=\frac{\sum {\left({\tilde{y}}_{i}-\bar{y}\right)}^{2}}{\sum {\left(y_{i}-\bar{y}\right)}^{2}},\;$$

(27) $$\bar{y}=\frac{1}{N}\sum_{i=1}^{N}y_{i},\;$$

where *N* is the number of training samples, $y_{i}$ is the actual value, and ${\tilde{y}}_{i}$ is the predicted value of the model. RMSE can be used to reflect the change amplitude of data. The smaller the value is, the higher the accuracy of the model prediction will be. MAE is not easily affected by outliers, and the smaller the value, the smaller the error. $R^{2}$ is used to evaluate the fitting effect of the model, and the value is (0, 1). The closer the value is to 1, the better the fitting effect of the model is.

#### 5.3.2. Results and Analysis of Optimal Parameter Combination of Model

**Figure 7:**
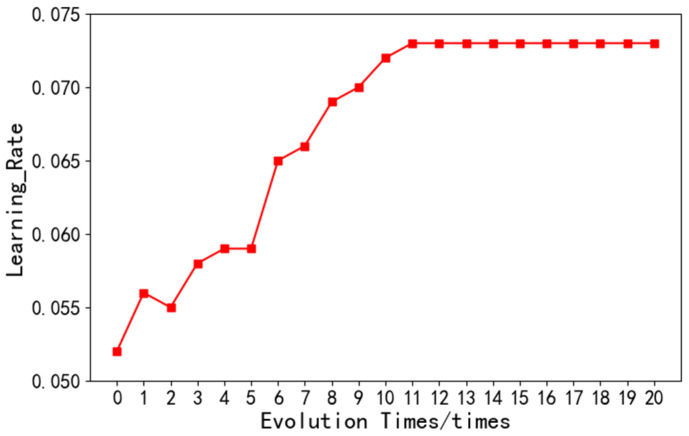
Learning rate of neural network.

**Figure 8:**
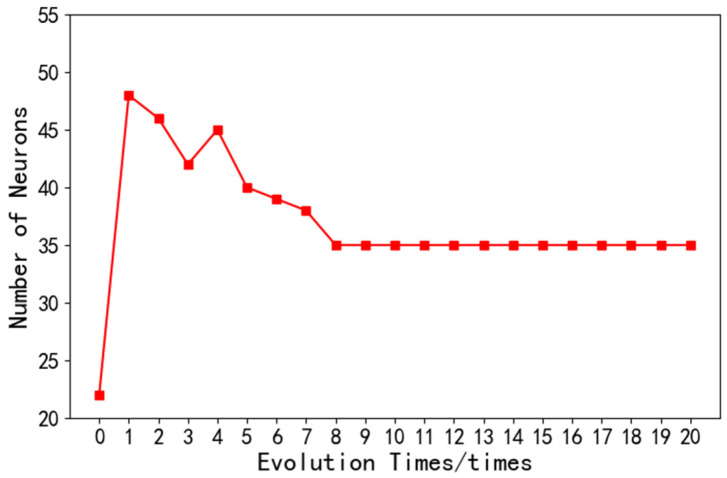
Data number of neurons.

**Figure 9:**
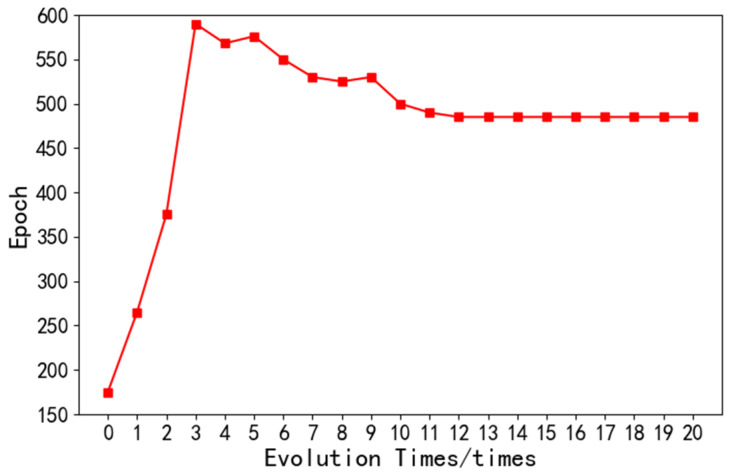
The epoch of neural network.

Hyperparameters have a significant influence on the deep neural network, which directly affects the quality of the model. In this experiment, it is verified that the selected hyperparameters are Learning_rate, the number of neurons, and the epoch. The specific selection is as follows:

(1) Learning rate: A large choice for learning rate will lead to the lowest loss, while a small choice will lead to the local optimum of the result. Therefore, an appropriate learning rate is crucial. As shown in Figure 7, with the iterative evolution of the PSO optimization algorithm, the learning rate stabilized at 0.073 in the 12th iteration of the optimization algorithm. Therefore, the learning rate selected by the model was 0.073.

(2) Several neurons: The number of neurons will affect the learning ability and network complexity of the model. Too many nodes will prolong the network training time, while too few nodes will lead to poor network performance. As shown in the Figure 8, after the ninth iteration of the optimization algorithm, the number of neurons was stable at 35.

(3) Epoch: Epoch means training the network model once with all the data in the training set. Through the continuous iteration of the neural network, the loss value can be minimized. As shown in the Figure 9, after the 11th iteration of the optimization algorithm, the number of iterations of the network model finally stabilized at 500.

### 5.4. Comparison and Analysis of Simulation Results of Different Algorithms

This experiment shows the prediction performance of AT-GRU by comparing it with the FARIMA model, the SVM model, and the GRU model.

FARIMA: Fractional Difference Autoregressive Moving Average Model [28], which is a self-similar model, can capture both long-term and short-term correlation characteristics of traffic data.

SVM (Support Vector Machine): It maps the feature vectors of instances to some points in the space [29]. The purpose of SVM is to draw a line to distinguish these two types of points, so that if there are new points in the future, this line can also make a good classification.

GRU: Gate Recurrent Unit [30], which is a variant network form of RNN neural network, can effectively capture the characteristics between long sequences and alleviate the phenomenon of gradient disappearance or gradient explosion.

**Figure 10:**
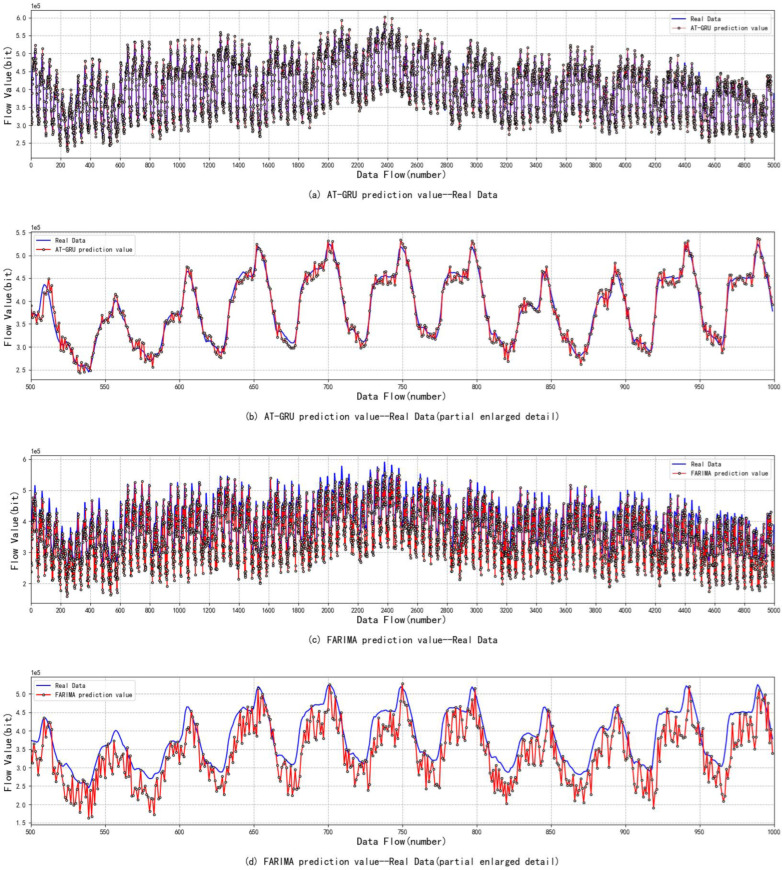

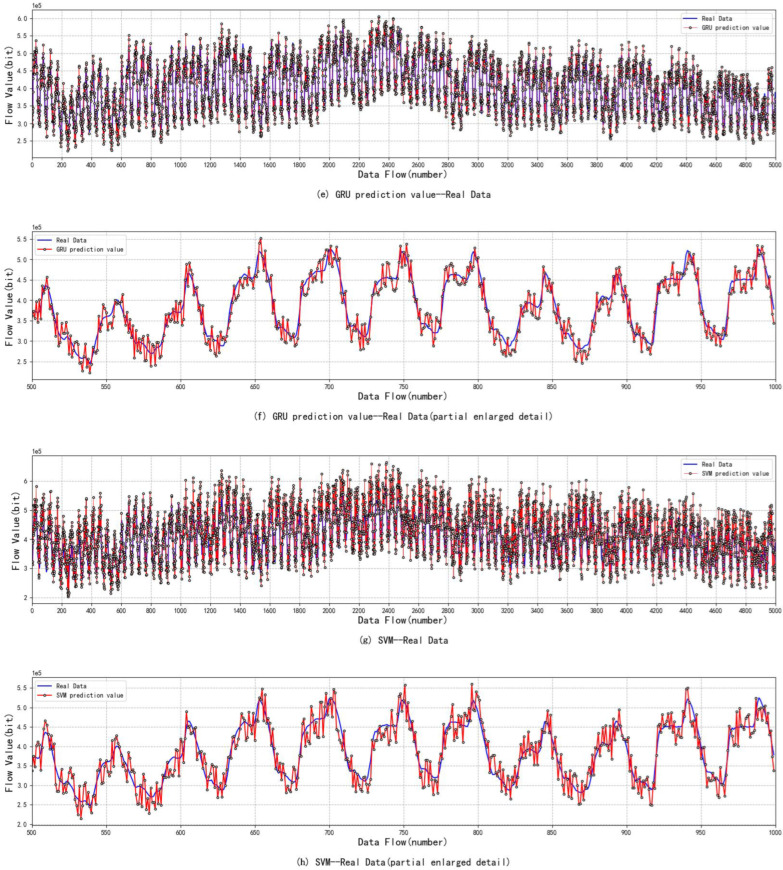
Comparison of prediction effects of different prediction models.

The above experiments show the comparison results of the prediction effects of various prediction models. In order to clearly express the experimental results, in Figure 10, (b), (d), (f) and (h) are partial enlarged images of (a), (c), (e) and (g) respectively. Simulation experiments show that all the above four prediction models have a certain prediction ability, but the AT-GRU algorithm proposed in this paper has the most obvious fitting effect. The AT-GRU network model can better reflect the complex characteristics of satellite network traffic data, and its prediction performance is better. Compared with other prediction models, the error is lower, with an MAE of 14.24, RMAE of 20.37, and $R^{2}$ score closest to 1. The FAR algorithm has the worst prediction performance, and the FARIMA traffic prediction model can only roughly predict the changing trend of traffic, but there is still a large error with the real traffic value. As FARIMA is a linear series model, it can only process short-time data series, but cannot fit complex nonlinear data, so the prediction error of this model is the largest. MAE was 33.73, RMAE was 42.20, and $R^{2}$ scored the lowest. The SVM algorithm can fit the real value well, but the overall traffic prediction effect has some errors. Because the SVR algorithm is mainly used to solve the linear regression problem of small samples, it cannot well reflect the complex characteristics of satellite network traffic. For the prediction of large samples, the error is large, and the model MAE is 22.68 and RMAE is 32.25. In this paper, the attention mechanism is introduced into the GRU network. Compared with the traditional GRU prediction, it can make full use of the target sequence, and the prediction effect is better than that of a single GRU neural network model. The prediction error MAE of a single GRU model is 19.49 and RMAE is 28.08.

**Figure 11:**
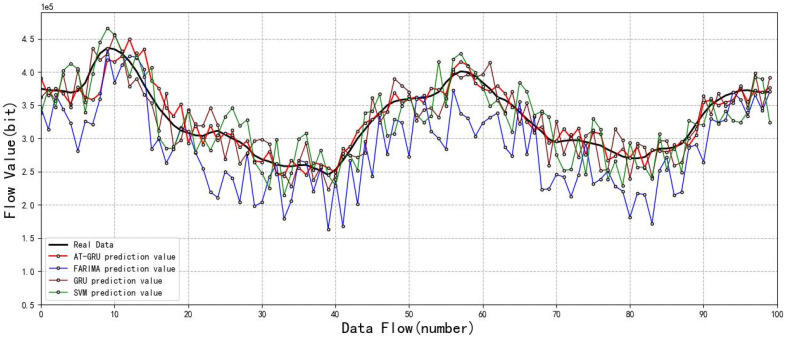
Model fitting effect.

As can be seen from Figure 11, AT-GRU has the best fitting effect and the lowest prediction error. The addition of an attention mechanism has promoted the prediction of GRU networks, which can improve the prediction accuracy of satellite network traffic to a certain extent and better meet the demand for satellite network traffic prediction.

### 5.5. Convergence Analysis

**Figure 12:**
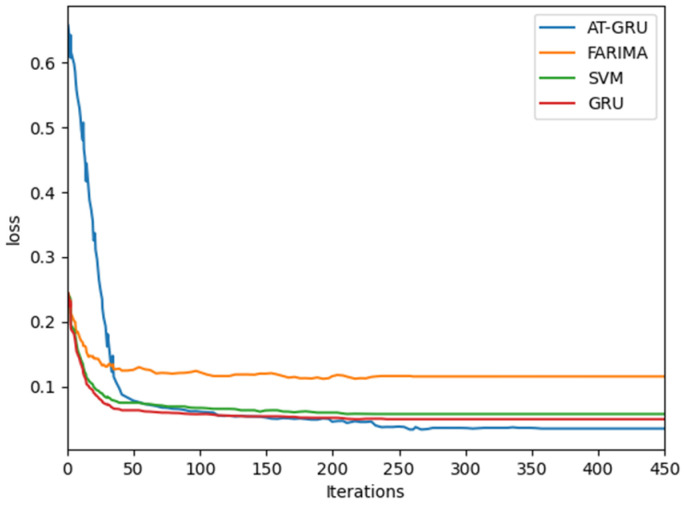
Model convergence performance diagram.

Figure 12 shows that the proposed method has obvious advantages in convergence speed, which shows that the model can learn data well.

### 5.6. Model Complexity Analysis

Time complexity and space complexity are two important indexes to measure an algorithm, which are used to indicate the amount of time increase and auxiliary space required by the algorithm in the worst state. In deep learning, the Flops value is usually used to explain the time complexity of the model, and the parameters of the model are used to explain the space complexity of the model. Based on the above description of model prediction steps, the time complexity of each model training is analyzed. The complexity of a neural network is usually represented by floating-point arithmetic number Flops. Based on the results in Table 2, we can clearly see that the Flops value of the scheme proposed in this paper is the lowest, so the complexity of the scheme proposed in this paper is the lowest. Meanwhile, according to the results of Figure 12, it can be seen that the loss value of the scheme proposed in this paper decreases the fastest and the loss value is the smallest. We can see that the complexity of the model after optimization is lower than that before optimization. Based on the analysis, it can be concluded that the complexity of the proposed scheme is obviously better than that of the comparison algorithms.

## 6. Summary and Prospect

### 6.1. Critical Analysis and Discussion

To improve the accuracy of satellite network traffic prediction, this paper proposes and verifies a satellite network traffic prediction method based on GRU neural network and attention mechanism. Based on GRU neural network, the attention mechanism is integrated to pay attention to the importance of traffic data and hidden state, which not only learns the time-dependent characteristics of input sequences but also mines the interdependent characteristics of data sequences, thus improving the accuracy of traffic prediction. At the same time, the PSO algorithm is used to determine the optimal hyperparameter combination of the model, which makes the model have higher prediction efficiency when forecasting traffic. In this paper, the effectiveness of the proposed method is illustrated by comparing it with several popular traffic prediction algorithms. In terms of average absolute error, the error of the proposed algorithm is 26.9% lower than that of the GRU model, 37.2% lower than that of the SVM model, and 57.8% lower than that of the FARIMA model. In terms of root mean square error, the proposed algorithm is 27.5% lower than the GRU model, 36.8% lower than the SVM model, and 51.7% lower than the FARIMA model. Furthermore, the goodness of fit of the new model is better than that of the comparison models. Simulation results show that the proposed method can improve the accuracy of satellite network traffic prediction, reduce the prediction errors, and have the best fitting degree with real traffic data.

### 6.2. Prospect

The model proposed in this paper has improved the prediction accuracy of satellite network traffic to a certain extent, but it still needs further improvement; more network noise will be introduced in the actual satellite network environment, which will affect the accuracy of network traffic prediction to a certain extent. In future work, we can further reduce the noise of network traffic and improve prediction accuracy.

## Figures and Tables

**Table 1:** Basic information on hardware.

Hardware	Value
CPU	Core i7-9700K
GPU	NVIDIA GeForce RTX 2080TI
Memory capacity	11 G
RAM	64 G
Disk capacity	2 TB

**Table 2:** Error comparison of different models.

Prediction Model	MAE	RMSE	*R* ^2^	FLOPS
GRU	19.49	28.08	0.8499	28.61 G
SVM	22.68	32.25	0.8459	28.06 G
FARIMA	33.73	42.20	0.7142	55.62 G
AT-GRU	14.24	20.37	0.9552	25.03 G

## Data Availability

The processed data required to reproduce these findings cannot be shared as the data also forms part of an ongoing study.
